# The Influence of Age and Physical Activity on Locomotor Adaptation

**DOI:** 10.3390/brainsci13091266

**Published:** 2023-08-31

**Authors:** Shawn Hiew, Leila Eibeck, Carine Nguemeni, Daniel Zeller

**Affiliations:** Department of Neurology, University Hospital of Würzburg, 97080 Würzburg, Germany; leila.eibeck@stud-mail.uni-wuerzburg.de (L.E.); nguemeni_c@ukw.de (C.N.); zeller_d@ukw.de (D.Z.)

**Keywords:** locomotor adaptation, walking, physical activity, exercise, aging, balance

## Abstract

Background: Aging increases individual susceptibility to falls and injuries, suggesting poorer adaptation of balance responses to perturbation during locomotion, which can be measured with the locomotor adaptation task (LAT). However, it is unclear how aging and lifestyle factors affect these responses during walking. Hence, the present study investigates the relationship between balance and lifestyle factors during the LAT in healthy individuals across the adult lifespan using a correlational design. Methods: Thirty participants aged 20–78 years performed an LAT on a split-belt treadmill (SBT). We evaluated the magnitude and rate of adaptation and deadaptation during the LAT. Participants reported their lifelong physical and cognitive activity. Results: Age positively correlated with gait-line length asymmetry at the late post-adaptation phase (*p* = 0.007). These age-related effects were mediated by recent physical activity levels (*p* = 0.040). Conclusion: Our results confirm that locomotor adaptive responses are preserved in aging, but the ability to deadapt newly learnt balance responses is compromised with age. Physical activity mediates these age-related effects. Therefore, gait symmetry post-adaptation could effectively measure the risk of falling, and maintaining physical activity could protect against declines in balance.

## 1. Introduction

Locomotor adaptation is an error-driven motor learning process used to alter spatiotemporal elements of walking [1]. This ability modifies walking patterns in response to different environmental demands, age-related changes in neural motor control or injury [2]. This skill is a critical ability for human beings, allowing them to navigate the world and move from one place to another safely.

Locomotor adaptive capacities can be probed experimentally by evaluating the changes in gait and balance parameters following an environmental perturbation. Difficulty adapting to new environments is linked to an increased rate of falls in older adults. Hence, measures of gait stability can predict risk of falls [3,4,5,6]. Disturbances to gait occur not only in disorders of the sensorimotor system, but also in biological or pathological processes that affect cognition such as aging or dementia [7]. Adaptive responses within the central nervous system (CNS) are believed to alleviate functional decline due to aging or neurodegenerative disorders [8,9,10]. Early modulation of adaptive capacities through specific training paradigms and/or pharmacological approaches targeting the underlying circuits may be suited to postponing the onset of motor impairments. Therefore, it is crucial to develop effective strategies to evaluate individual capability to successfully compensate for the clinical impact of aging and neurodegeneration.

The locomotor adaptation task (LAT) on a split-belt treadmill (SBT) has already proven to be a useful tool to probe gait adaptation. The general ability to adapt has been shown to be preserved in different populations with neurological decline including people with multiple sclerosis (MS) [11,12], people with Parkinson’s disease [13] and healthy older adults [2]. However, the age-related changes in the neural control of locomotor adaptation remain unclear, and several contradictory analyses exist in the literature. Studies have shown that older adults exhibit a magnitude of adaptation similar to young adults [13,14,15,16], but the rate of predictive (or feedforward) adaptation is decreased [17]. Contrary to reactive (or feedback) adaptation, which refers to immediate postural reactions to a perturbation reflected in changes to intralimb parameters, predictive adaptation (which is an implicit change driven by motor performance prediction error) requires time and prolonged exposure to a perturbation, and it is reflected in interlimb parameters, such as step length, the distance between the feet during a heel strike [2]. The introduction of a perturbation in the adaptation phase of SBT walking leads to an initial exaggerated distance between feet during the heel strike of the slow foot in comparison to the fast foot, resulting in a marked asymmetry in the step length of the two legs. The degree of asymmetry is reduced over the course of the adaptation period and is exaggerated in the opposite direction when the belts are tied once again during the post-adaptation period, reflecting aftereffects, which ultimately wash out over time. The poorer rate of predictive adaptation in older adults found by Bruijn et al. [18] has not been reproduced in other studies, bringing into question this age-based effect [19,20,21]. However, in the latter studies, the cohorts of older adults were younger (55.3 ± 2.91 [20]; 67.8 ± 5.8 [21]) than in the study by Bruijn and colleagues (73.1 ± 4.7 [17]). Hence, it is likely that the age-related difference may only become prominent after 70 years of age. A more in-depth evaluation of locomotor adaptation across the human lifespan is therefore necessary to draw a conclusion as to what point of age affects locomotor adaptation ability as well as about the mediating effects of other factors. 

Aging is indeed complex, and many other factors are likely to play a role in its effects on locomotor adaptation. Cognitive processes have been suggested to mediate motor adaptation, with evidence indicating that older adults adapt more poorly when a simultaneous cognitive task is introduced [22,23]. Moreover, the cognitive reserve, which refers to individual differences in cognitive abilities to adapt to age-related changes in healthy and pathologic contexts such as aging or neurodegenerative brain disease, is probably an important factor as well [18]. In addition to cognition, the amount of physical activity that an aging adult engages in is also likely to mediate locomotor adaptation. Thus, when matched for physical activity levels, the age-related differences in adaptation rates diminished [24]. Accordingly, it comes as no surprise that physical activity levels have been linked to lower mortality and decreased risk of falling [25,26]. The impact of physical activity has drawn significant interest in the last few years due to its probable influence on disease onset and progression, as well as its therapeutic potential [27,28]. For example, training on an SBT has been demonstrated to lead to long-lasting improvements in gait stability and endurance in people with MS [29]. Physical activity may support the building of a motor reserve, protecting against age- and disease-related decline, and provided sufficient adaptive capacity, physical activity can be a promising intervention. Yet, to date, there has been no thorough investigation into the effect of physical activity on locomotor adaptation.

The ability to adapt gait stability and balance to environmental challenges during locomotion is crucial to reduce the risk of falls and injuries and has been shown to be affected by aging [20]. Previous studies evaluating the effect of aging on locomotor adaptation have not thoroughly considered the influence of lifestyle factors, including physical activity. Hence, the present study will assess locomotor adaptation across the adult lifespan and its associations with lifestyle factors. We, therefore, aim to (i) identify the patterns of change in gait stability and balance during a locomotor task in adults across the lifespan and (ii) to verify whether age, cognitive reserve and physical activity throughout life are associated with the adaptation of gait stability to split-belt walking. We hypothesize that gait-line length symmetry, as a measure of balance, at the start of perturbed walking and during the return to unperturbed walking will decline with age, an indicator of poorer adaptive control of balance. The rate of gait-line length adaptation will also decrease with age. Cognitive reserve and lifelong physical activity will mediate the age-related decline in adaptive control of balance. To the best of our knowledge, no other study has considered the interaction between cognition, physical activity and age regarding locomotor adaptation, nor has any other study evaluated this interaction across the lifespan.

## 2. Materials and Methods

### 2.1. Participants

The study conformed to the principles of the Declaration of Helsinki and was approved by the local ethics committee of the Medical Faculty at the University of Würzburg under the number 252/21. All methods were performed in accordance with the relevant guidelines and regulations. All participants gave their written informed consent and were naive to the purpose of the study.

An a priori power analysis was performed using G*Power 3.1.9.7, based on a related study reporting R = −0.68 for the correlation between age and gait-line length symmetry on a locomotor adaptation task [17]. Based on this, but with a more conservative approach, we expected an effect size of about 0.5. With α = 0.05, a minimum sample of N = 23 was required.

In total, 30 participants (15 females, age range: 20–78 years) without neurological or psychiatric disorders were recruited for this study. Prior to recruitment, they received an email detailing the experimental timeline, the exclusion criteria and the measures, including the questionnaires. Furthermore, they were asked about pre-existing health conditions and medications. 

Exclusion criteria were pregnancy, centrally acting drugs, pain or other conditions which may preclude performance of the behavioral tasks, including dementia and mild cognitive impairment, as well as inability to perform the locomotor adaptation task (LAT) on a split-belt treadmill (SBT). 

We used the Beck Depression Inventory (BDI) to assess depression [30], the Montreal Cognitive Assessment (MoCA) for evaluating cognitive function [31], the Frontal Assessment Battery (FAB) [32] to evaluate frontotemporal function and the Cognitive Reserve Questionnaire (CRQ) [33] for quantifying the cognitive reserve. The history of physical activity was evaluated using the HISTPAQ questionnaire [34].

### 2.2. Experimental Design 

The single-session study used a correlational design. Participants underwent the clinical tests (MoCA and FAB) and completed the different questionnaires (BDI, CRQ and HISTPAQ) before starting the experiment. Their fastest possible walking speed was determined using the timed 25 ft walking test. This walking test was performed twice, and the mean speed was used to determine the speed of the treadmill belts for the LAT. The 34 min LAT was performed once during the study.

### 2.3. Split-Belt Treadmill Paradigm

Subjects participated in an LAT on an SBT (Woodway USA, Waukesha, WI) with belts moving together (TIED) or at different speeds (SPLIT). The speed of the treadmill was determined for each participant individually based on their speed on the 25FWT. The mean speed of the two trials was used as the fast speed for the LAT on the SBT, the slow speed was defined as one third of the fast speed, and the average speed was the mean of the slow and fast speeds.

**Figure 1:**
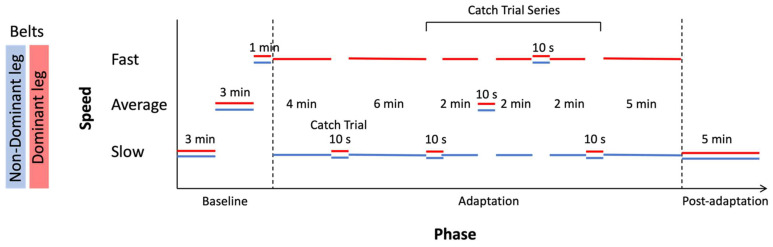
Split-belt treadmill paradigm. The two belts are represented by blue for the non-dominant leg and red for the dominant leg. The speeds of the two belts during the locomotor adaptation task (LAT) are presented for each phase. At baseline, the belts are tied at slow and then at average speed for 3 min, and finally at a fast speed for 1 min. After that, the belts split, and the belt of the dominant leg moves at the fast speed while the non-dominant belt moves at the slow speed during the adaptation phase. During this phase, the belts are tied again for 4 catch trials, for 10 s each at the three different speeds. Finally, during the post-adaptation phase, both belts are tied at the slow speed.

Details of the SBT paradigm can be viewed in Figure 1. Following the familiarization phase, participants walked at different baseline speeds with both belts moving at the same slow speed (in TIED condition) for 3 min, then at average speed for 3 min and finally at fast speed for 1 min. The adaptation phase followed immediately, lasting 10 min, during which the belts moved with a slow/fast speed ratio of 1:3 [35] in the SPLIT condition. The side of the fast belt for each participant was defined by the participant’s preferred leg when spontaneously kicking a ball [36]. Because adaptive mechanisms to reduce the initial limping and gait instability induced by introducing a perturbation in the SPLIT condition have been observed to appear within the first few minutes after perturbation onset [37,38], the adaptation phase was disrupted at 4 min with a 10 s trial where participants walked in TIED condition. Because people have been demonstrated to adapt within the first four minutes of the adaptation, a series of catch trials at the three different speeds (slow, average and fast) were introduced in a random order to induce surprise and thus maximize the effects of the split-belt adaptation phase [29]. The 10 min adaptation phase was followed by a series of catch trials where participants walked for 15 s in the TIED condition at one of three predefined speeds (slow, average or fast), after which the subjects readapted for 2 min in the SPLIT condition. A total of four catch trials were performed, where the first and last catch trials were always at the slow speed. The second and third catch trials were at the fast and average speeds, with the order of the two speeds randomized and counterbalanced across subjects. After the last catch trial, a readaptation phase lasting 5 min in the SPLIT condition followed [39]. Finally, a 5 min post-adaptation phase in the TIED condition at slow speed [40] was performed. Participants each wore a safety harness and were oriented with one leg on each belt of the treadmill.

### 2.4. Modified Retrospective Physical Activity Survey

A modified retrospective physical activity survey [34] was used for quantifying the physical activity behavior of the participants. This survey divides the lifespan into 4 periods (14–21, 22–34, 35–50, 50+ years), and participants report their different regular physical and sports activities for each period in hours per year. To quantify their activity in the different periods, a kcal amount for each period was calculated by multiplying the hours of an activity with the required kcal per h listed for the specific sport. The required kcals were assessed by dividing the sports into 3 categories (300, 450 and 600 kcal/h needed) [41]. The kcal amounts for each period were then summed and divided by the number of weeks in this period to obtain a kcal/week measure for each period, later referred to as the history of physical activity (HISTPAQ). 

### 2.5. Data Collection 

Gait parameters were recorded using special insoles consisting of 13 pressure sensors and a 3D acceleration sensor (Moticon Insoles, Munich, Germany). These insoles have been validated in the clinical setting [42,43]. The gait-line length, that is, the path of the anterolateral–posterolateral center of pressure (CoP) under the foot during one ground contact phase, was collected for each step to complete the motion analysis. This measure was also previously used by Nguemeni and colleagues (2020) [11].

### 2.6. Data Analysis

Gait data were grouped into baseline, adaptation and post-adaptation phases. Mean gait-line lengths (mm) for the slow and fast foot were extracted for the following phases:

- Baseline (B): the last six strides of the baseline phase;
- Early Adaptation (EA): the first six strides of the adaptation phase;
- Mid-Adaptation (MA): the last six strides before the 10 s TIED trial;
- Late Adaptation (LA): the last six strides of the adaptation phase;
- Early Post-adaptation (EPA): the first six strides of the post-adaptation phase;
- Late Post-adaptation (LPA): the last six strides of the post-adaptation phase.

The gait-line length symmetry for each of the six phases was calculated as follows:

$$\mathrm{Gaitline}\mathrm{length}\mathrm{symmetry}=\frac{\mathrm{Gaitline}\mathrm{lengthfast}-\mathrm{Gaitline}\mathrm{lengthslow}}{\mathrm{Gaitline}\mathrm{lengthfast}+\mathrm{Gaitline}\mathrm{lengthslow}}$$

We evaluated the magnitude of adaptation, defined as the difference between the gait-line length symmetry at mid-adaptation, the phase at which we postulate that all participants have fully adapted, and early adaptation. The magnitude of deadaptation was calculated as the difference between the mean gait-line length symmetry at late post-adaptation and early post-adaptation. Finally, the magnitude of aftereffects was taken as the difference between the mean gait-line length at early post-adaptation and at baseline.

In order to assess the rate of adaptation and deadaptation, the adaptation and deadaptation plateaus were first defined by identifying the first step after the start of adaptation and deadaptation, where the subsequent 5 steps fell within a plateau range, defined as the mean ± the standard deviation of the last 30 steps of the adaptation or deadaptation phase. The rate was then calculated as follows:

$$rate=\frac{\mathrm{Gaitline}\mathrm{length}\mathrm{plateau}\mathrm{step}-\mathrm{Gaitline}\mathrm{length}\mathrm{first}\mathrm{step}}{\mathrm{No.}\mathrm{of}\mathrm{steps}\mathrm{to}\mathrm{plateau}}$$

The history of physical activity for the age period of 14–21 years, measured in kcal/week, was taken as HISTPAQ_early_, while the history of physical activity of each participant in their present age period was taken as HISTPAQ_recent_.

### 2.7. Statistics

To evaluate the effects of the six different phases on gait-line length symmetry, repeated measures analyses of variance (ANOVA) with Tukey post hoc comparisons were performed with the phase as the within-subjects variable. Data were tested for normality and sphericity using the Shapiro–Wilk test and Mauchly’s test, respectively. Non-parametric statistical tests, i.e., Friedman’s test with Dunn’s post hoc comparisons, were used when the data were found to violate the assumption of normality, and a Greenhouse–Geisser correction was applied to adjust for lack of sphericity where applicable.

To evaluate the relationship between age and gait-line length symmetry at the different phases, two-tailed Spearman’s correlations were computed in JASP (Jeffrey’s Amazing Statistics Program, The JASP Team, 2020). One-tailed Spearman’s correlations were computed to assess the associations between physical activity, CRIq and gait-line length symmetry. Where age correlated with gait-line length symmetry, the associations between physical activity, CRIq and gait-line length symmetry were evaluated using Spearman’s partial correlations with age as a covariate. Spearman’s correlations were also performed to investigate the relationships between age and rates and magnitudes of adaptation, deadaptation and aftereffects. One-tailed Spearman’s correlations were computed to assess the associations between physical activity, CRIq and rates and magnitude of adaptation, deadaptation and aftereffects. Where age correlated with rates and magnitudes of adaptation, deadaptation and aftereffects, the associations between physical activity and CRIq were evaluated using Spearman’s partial correlations with age as a covariate.

## 3. Results

### 3.1. Demographic and Psychometric Data

The 30 participants were aged between 20 and 78 years (48.53 ± 16.60; males: N = 16). Two-tailed Spearman’s correlations revealed no significant correlations between age and Beck Depression Inventory scores (BDI [30]), Montreal Cognitive Assessment (MoCA [31]) scores, Frontal Assessment Battery (FAB [32]) scores or adolescent and most recent physical activity (HISTPAQ_early_ and HISTPAQ_recent_ [33]) scores. However, significant positive correlations between age and time on the 25 ft walking test (25FWT) as well as cognitive reserve index questionnaire (CRIQ [34]) score were revealed. Participants’ demographic characteristics and their correlations with age are summarized in Table 1.

### 3.2. Gait Symmetry during the Locomotor Adaptation Task

**Figure 2:**
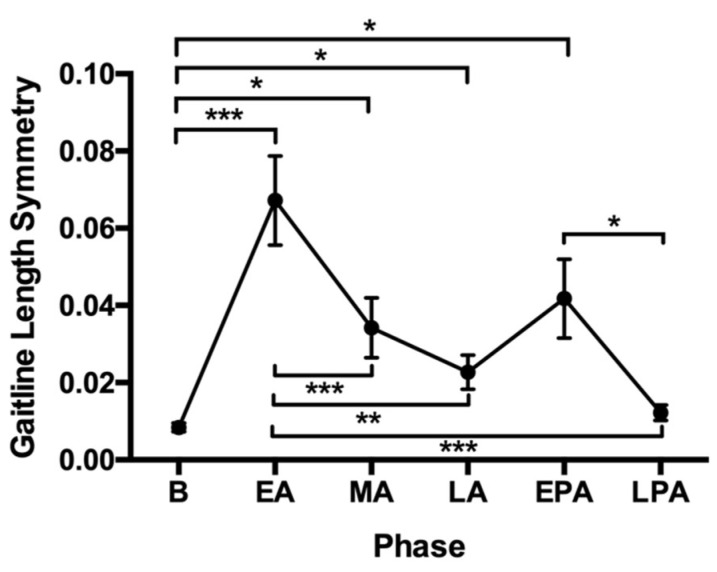
Gait-line length symmetry at the different phases. Abbreviations: B, baseline; EA, early adaptation; EPA, early post-adaptation; LA, late adaptation; LPA, late post-adaptation; MA, mid-adaptation. * *p* < 0.05; ** *p* < 0.01; *** *p* < 0.001.

All participants were able to complete the walking task on the SBT. Repeated measures analyses of variance (ANOVA) revealed a significant effect of phase on gait-line length symmetry (F (2.54, 73.69) = 11.60, *p* < 0.001; Figure 2). Tukey post-hoc comparisons revealed that the gait-line length was more symmetrical at baseline than in all other phases aside from late post-adaptation (*p* < 0.050). Gait-line length symmetry improved during the adaptation phase in mid- and late adaptation compared to early adaptation (*p* < 0.001). Finally, the asymmetrical gait-line length improved at late post-adaptation compared to early post-adaptation (*p* < 0.050).

### 3.3. Correlation between Age and Locomotor Adaptation

**Figure 3:**
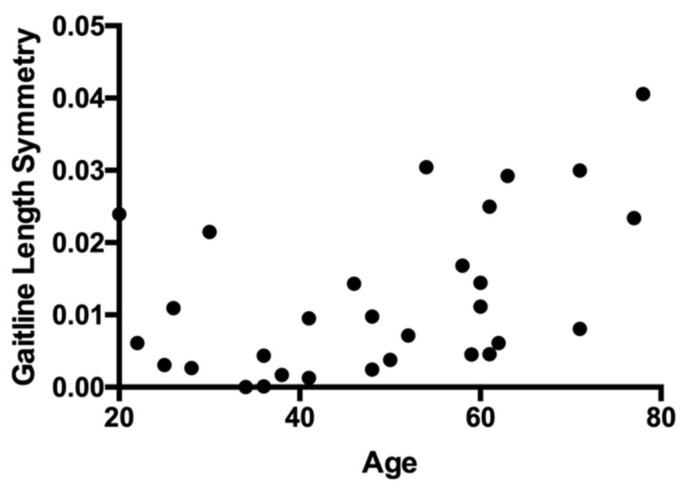
Gait-line length symmetry at late post-adaptation plotted against age.

Spearman’s correlation revealed a significant positive correlation between age and gait-line length symmetry at late post-adpatation (r_s_(28) = 0.48, *p* = 0.007, Figure 3). No significant correlations were found between age and gait-line length symmetry at any other phase (*p* > 0.05). Age did not correlate with the magnitude and rate of adaptation and deadaptation or the magnitude of aftereffects (*p* > 0.05).

### 3.4. Correlation between Physical Activity and Locomotor Adaptation

One-tailed Spearman’s correlation tests revealed a significant correlation between HISTPAQ_recent_ and rate of deadaptation (r_s_(28) = −0.39, *p* = 0.017). One-tailed Spearman’s partial correlations with age as a covariate reveal a significant correlation between HISTPAQ_recent_ and gait-line length at late post-adaptation (r_s_(27) = −0.33, *p* = 0.040). No other significant correlations were found between HISTPAQ_recent_ and gait-line length symmetry, the rate and magnitude of adaptation and deadaptation or the magnitude of aftereffects. Additionally, no significant correlations were found between HISTPAQ_early_ and gait-line length symmetry, the rate and magnitude of adaptation and deadaptation or the magnitude of aftereffects.

CRIq was not found to correlate with gait-line length symmetry, the rate and magnitude of adaptation and deadaptation or the magnitude of aftereffects. 

## 4. Discussion

The present study examined locomotor adaptation in adults across the lifespan, attempting to elucidate the effect of aging on the adaptation of balance responses during a locomotor adaptation task. Moreover, we aimed to probe the influence of lifestyle factors, namely the cognitive reserve and lifelong physical activity, on individual adaptive capacity. Our findings provide further support for the idea that older adults retain the ability of locomotor adaptation. However, we demonstrate that the gait-line length asymmetry post-adaptation increased with age, suggesting a poorer deadaptation of newly learned balance responses after a perturbation has been removed. This observation is mediated by recent physical activity, whereby higher levels of recent physical activity lead to a more symmetrical gait-line length post-adaptation.

### 4.1. Locomotor Adaptation Is Preserved in Older Adults

In our study, we were unable to find an age-related effect on locomotor adaptation ability. All subjects in the study were able to adapt their balance responses to a perturbation. One explanation is that the age-related decline in walking speed, as evidenced by the strong positive correlation between time on the 25FWT and age, was controlled for by individualizing the belt speeds. As such, the speeds of the belts were equally challenging for all participants regardless of age. The observed effect of age on the ability to adapt in some previous studies may have been driven by individual walking speed [17,18]. However, it must be noted that only four of our participants were over the age of 70, unlike other studies that have included larger numbers of older adults [17]. Nevertheless, our findings seem to be in line with studies whose older cohorts match the age range of ours, showing no age-related effect on locomotor adaptation [19,20,21].

### 4.2. Older Adults Show Longer-Lasting Aftereffects

While age did not seem to affect the adaptation phase, it was strongly associated with the washout phase, such that older adults retained more aftereffects when a perturbation was removed. This age-related observation could be explained by an increase in motor perseveration with age, i.e., the declining ability to switch between motor tasks. Indeed, older adults have been demonstrated to be poorer at switching from split-belt to overground walking [44].

While several studies have shown reduced aftereffects, such as post-adaptation asymmetry, combined with a decreased rate of deadaptation in the elderly, the older adults in our study showed significantly reduced symmetry at the late post-adaptation phase, where younger adults had returned to relatively more symmetrical gaits, albeit with similar rates of deadaptation [17]. One interpretation is that the initial asymmetry induced when the belts return to the same speed is reduced to a lesser extent in older adults. A plateau may occur earlier in the deadaptation process for older adults, preserving their rate of deadaptation, but this deadaptation may be incomplete by the end of the 5 min washout phase in this study. This could be attributed to the different priorities of older adults compared to younger adults, with the former prioritizing stability over energy optimization, and the latter vice versa [45,46]. As such, younger adults, having learnt from a series of catch trials, are able to deadapt completely, while in the older adults, some remaining aftereffects are preserved in anticipation of another perturbation to adapt to. 

Alternatively, older adults may be poorer at responding to cues to switch motor patterns. This has been demonstrated in a stroke population where the symmetry induced by split-belt walking, relative to their naturally asymmetrical gait, was retained and transferred when returning to overground walking [47]. A practical implication of our finding is that measurements of gait symmetry at the late post-adaptation phase may be sufficiently sensitive to aging and gait disturbances and could be capitalized upon for preventative and rehabilitative therapies in aging adults and adults with gait disturbances.

### 4.3. Physical Activity Mediates Age-Related Effects Post-Adaptation

Physical activity has been linked to improved motor learning and memory as well as an improved ability to switch between motor patterns [48]. Additionally, physical activity is neuroprotective and can therefore preserve the integrity of brain regions which are crucial for adaptation. For instance, physical activity can reduce age-related declines in cortical tissue density in the frontal, temporal and parietal cortices significantly [49], increase the hippocampal volume and therefore improve cognition [50], increase the level of trophic factors in plasma and serum in persons with neurodegenerative disorders [51] and promote neuroplasticity in persons with mood disorders [52].

The findings of the present study support the notion that current physical activity levels influence the ability to deadapt in adults across the lifespan. While physical activity levels did not appear to influence the rate of adaptation, greater recent physical activity levels mediated the age-related effect on gait asymmetry post-adaptation. Therefore, the new motor program formed may have been strengthened over the course of the adaptation phase, and unlearning the new program is a challenge that is made easier by higher physical activity levels. Such a hypothesis is in line with findings by Reisman et al. [47], whose stroke patients showed similar adaptive abilities but held onto the new walking pattern when they returned to overground walking. While learning a new motor program is a crucial part of locomotor adaptation ability, it is equally crucial to return to the old program when a perturbation is removed. However, it remains a debate as to whether this happens via unlearning or the masking of the new program by a competing motor memory [53,54]. Hence, the ability to revert to the old motor program during unperturbed walking, whether by unlearning or by using a competing motor memory, is an indicator of an individual’s capacity for locomotor adaptation.

The ability to unlearn is part of a healthy motor program that relies on the cognitive ability to detect environmental change. In the case of unlearning, physical activity may have preserved this cognitive ability, promoting a more complete unlearning of the new motor program. In the case of a competing motor program, it would be reasonable to expect individuals engaging in higher levels of physical activity to also spend more time walking, therefore having a stronger memory of unperturbed walking that is capable of competing with the new motor program. Additionally, switching between motor programs is a higher-order cognitive function that typically declines with age, an ability preserved by higher brain function with increased physical activity [15]. It is likely, for this reason, that the SBT is an effective tool in improving gait asymmetries post stroke: because affected individuals would be engaging in less physical activity after the stroke, they benefit from the longer-lasting aftereffects [35]. Higher physical activity levels in older adults may therefore reduce the effect of aging, reflected by less aftereffects, resembling the state of the younger adults at the end of the washout phase. In accordance with this line of thought, older adults without cognitive impairment also tend to have higher levels of physical activity compared to older adults with cognitive impairment [55].

Because current physical activity levels mediated the age-related effect on gait symmetry post-adaptation, a motor reserve may play a role compensating for the effects of aging. However, the direction of this link remains elusive. Either the motor reserve acts as a buffer against the effects of age, or individuals better able to achieve a more symmetrical gait are more likely to engage in higher amounts of physical activity. To confirm this hypothesis, a sample with a wider range of physical activity levels and a more challenging LAT protocol, for example, one with an additional cognitive secondary task, are necessary. Dual tasking would not only be more challenging but more meaningful, as it simulates perturbed walking in the real world where distractions are present, and where one performs other important tasks such as navigation and crossing roads. 

The present results suggest that while the ability to adapt is preserved in older adults, aging may compromise the ability to deadapt learnt balance responses after a perturbation is removed and that this age-related effect is mediated by physical activity. Gait asymmetry at the late post-adaptation phase is sensitive to the effects of aging and may reflect physical activity levels, which are indicators of a potential motor reserve. 

### 4.4. Limitations and Future Directions 

In the present study, we had relatively few subjects that were over 70 years, the age at which most age-related effects on gait become most apparent, and the lack of variability in both cognitive abilities and physical activity levels among our subjects makes it difficult to truly pin down the age-related effect and the weight of cognition and physical activity factors in locomotor adaptation. Nevertheless, we identified a clear link between age and locomotor adaptation and the mediating role of physical activity. We showed that older adults retain more aftereffects during deadaptation, an effect that is mediated by recent physical activity levels. Potentially, a motor reserve may provide resources to compensate for age-related declines in motor task-switching. The link found in our group of relatively active adults further suggests that physical activity levels do influence gait motor task-switching. To take it a step further, this age- and physical activity-sensitive measure could shed light onto an individual’s ability for learning and switching motor programs, and possibly on their motor reserve. A larger sample of young and old adults with more variable physical activity levels is needed to confirm this association and to uncover additional links between physical activity and locomotor adaptation. Furthermore, in the present study, we did not acquire brain morphometric data and are unable to confirm whether the influence of physical activity on gait symmetry is due to a neuroprotective effect, therefore preserving the locomotor centers of the brain against aging. Future studies should therefore investigate the link between physical activity and the volume of the locomotor regions. 

Locomotion is no doubt a cognitively demanding task, and with a lack of variable cognitive abilities and cognitive reserves in our sample, a link between cognition and locomotor adaptation across the lifespan may have been occluded. A sample with a wider range of cognitive abilities will be necessary to evaluate the role of cognition in locomotor adaptation across the lifespan.

Finally, the measures of cognitive reserve and physical activity used in this study are self-reported, so they undoubtedly lack the accuracy of objective measures. However, accurate recording of parameters such as physical or cognitive activities in the daily life of individuals remains a challenge. While wearable technologies such as activity monitors, which have already become popular, may allow quantitative evaluation of present physical activity levels, retrospective assessment of physical activity, e.g., during youth, remains a matter of questionnaires. Nevertheless, due to the overall positive association between physical activity levels and skills such as motor learning, as well as its neuroprotective role, its positive influence on gait symmetry in the locomotor adaptation task described here is likely true.

## 5. Conclusions

Locomotor adaptation is a crucial skill that is preserved over the course of aging. Nevertheless, older adults are at greater risk of falling, as their adaptive balance responses are compromised with age. Aging leads to an increase in motor perseveration, which may be compensated for by physical activity providing a motor reserve. The locomotor adaptation task is therefore a useful tool for assessing the risk of falls in older adults and adults with restricted physical activity. Post-adaptation gait symmetry is sensitive to age, and this effect is mediated by physical activity. Additional studies with a larger sample of older and younger adults, with more variable physical activity levels and cognitive abilities, are warranted to confirm the influence of these factors on locomotor adaptive capacity. In conclusion, the aftereffects in the LAT may be a useful indicator of individual locomotor adaptive capacity.

## Figures and Tables

**Table 1:** Demographic characteristics of the participants and their correlation with age.

Measure	Mean [Range]	R_s_	*p*
BDI	4.3 ± 3.89 [0–14]	0.15	0.429
FAB	17.47 ± 0.78 [15–18]	0.24	0.202
MoCA	28.27 ± 1.62 [25–30]	−0.33	0.071
HISTPAQ_early_ (kcal/wk)	2425.83 ± 2432.93	−0.14	0.474
HISTPAQ_recent_ (kcal/wk)	2566.40 ± 1825.26	−0.07	0.705
**25FWT (s)**	**3.99 s ± 0.58**	**0.58**	**0.001**
**CRIQ**	**118.07 ± 18.50**	**0.83**	**<0.001**

Abbreviations: BDI, Beck Depression Inventory; CRIQ, Cognitive Reserve Index Questionnaire, FAB, Frontal Assessment Battery, HISTPAQ_early_, early history of physical activity; HISTPAQ_recent_, recent history of physical activity; LPA, late post-adaptation. Note: text **in bold** represents measures with significant correlations (*p* < 0.05).

## Data Availability

The datasets analyzed during the current study are available from the corresponding author on reasonable request.
